# Evaluating a neonatal public health education and empowerment programme to reduce infant mortality risks: insights from parents and facilitators in the midlands, UK

**DOI:** 10.3389/fgwh.2026.1843810

**Published:** 2026-07-22

**Authors:** Olufisayo Olakotan, Samuel Oluwatobi Atiku, Gregory Maniatopoulos, Nicola O’Brien, Thillagavathie Pillay

**Affiliations:** 1 Department of Neonatology, Women and Children’s Directorate, University Hospitals Leicester NHS Trust, Leicester, United Kingdom; 2 School of Digital, Technology, Innovation and Business, University of Staffordshire, Stafford, United Kingdom; 3 Research Data and Systems Manager, Aston University, Birmingham, United Kingdom; 4 School of Business, University of Leicester, Leicester, United Kingdom; 5 School of Psychology, Northumbria University, Newcastle upon Tyne, United Kingdom; 6 Faculty of Science and Engineering, University of Wolverhampton, Wolverhampton, United Kingdom; 7 College of Health Sciences, University of Leicester, Leicester, United Kingdom

**Keywords:** health inequalities, infant mortality, parent education, parental empowerment, preterm infants, STORK programme

## Abstract

**Introduction:**

Infant mortality rates in the Midlands are among the highest in the UK, with regional and ethnic disparities highlighting persistent inequalities, and risks greatest for infants born preterm. This calls for inclusive educational programmes that support diverse families in improving infant safety. The STORK (Supportive Training Offering Reassurance and Knowledge) parent education and empowerment programme, which includes a digital application to support learning and reinforce key messages, was developed to equip parents, families and carers with practical knowledge and skills to reduce key risks associated with infant mortality. This study evaluates the perceived usefulness of the STORK programme among parents of preterm infants in reducing risks associated with infant mortality and explores the future implementation of the programme in terms of programme facilitators' barriers and facilitators to programme delivery.

**Methodology:**

A descriptive qualitative study employing semi-structured interviews was conducted at University Hospitals of Leicester (UHL), UK. Participants were recruited between June and December 2024 and met inclusion criteria based on recent or prior engagement with the STORK programme. Interviews were recorded, transcribed verbatim, and analysed thematically using Braun and Clarke's six-step approach.

**Results:**

Sixteen parents who had received the programme and three STORK facilitators participated in the study. Parents reported that the STORK programme enhanced their confidence and preparedness in infant care, particularly in areas such as emergency response, safe sleep practices, and infection prevention. They expressed a preference for hands-on, early, and flexible delivery of the programme. The app was valued as a reference tool, though it was infrequently accessed following programme delivery. Facilitators highlighted the programme's positive impact but identified resource shortages and the need to further culturally tailor the STORK programme to better meet the needs of diverse populations as key challenges to effective delivery.

**Conclusion:**

This evaluation underscores the importance of culturally, parent-focused education in addressing persistent disparities in infant mortality. Insights from families and facilitators provide valuable guidance for refining and scaling education and empowerment programmes, helping to optimise reach, engagement, and impact across diverse healthcare settings.

## 1 Introduction

Infant mortality, or the death of a live-born baby within their first year of life, is one of the most important indicators of a nation's health and wellbeing (1). In England, the infant mortality rate was 3.9 deaths per 1,000 live births for eight consecutive reporting periods, but it increased to 4.1 per 1,000 in 2021–2023 (2). Regional disparities were evident: the West Midlands had a rate of 5.9 per 1,000 live births, double that of the South West, which reported 2.9 per 1,000 (2). In Leicester (East Midlands), the infant mortality rate was 7.7 per 1,000 live births, significantly higher than the national average of 4.1 per 1,000 during the same period. Significant disparities in infant mortality rates were also reported, with 6.8 per 1,000 for Black infants, 5.7 per 1,000 for Asian infants, and 3.1 per 1,000 for White British infants (3). Infant deaths are concentrated in the neonatal period, where the leading causes include immaturity-related conditions/prematurity, congenital anomalies and antepartum infections; in 2023, immaturity-related conditions accounted for 51.7% of neonatal deaths, while congenital anomalies and antepartum infections together accounted for a further 40.2% (4, 5). Although not all causes of infant death are preventable, several associated risk factors are modifiable, including lack of breastfeeding, maternal smoking, unsafe sleep practices, domestic abuse and barriers to accessing health information, some of which are addressed through STORK (Supportive Training Offering Reassurance and Knowledge) topics (6–9).

Public health educational interventions and programmes play a vital role in increasing parental knowledge and improving infant health outcomes (10–12). A well-documented and widely recognised example is the “Back to Sleep” campaign in the 1990s, which educated parents on placing babies on their backs to sleep. This simple message led to an 81% reduction in sudden unexpected death in infancy (SUDI) rates in the UK over the following years (13). In contrast, in the United States, where healthcare structures and safe sleep recommendations differ, a statewide hospital-based initiative demonstrated that providing educational materials such as sleep gowns, board books, and bassinets to Medicaid families improved adherence to recommended safe sleep practices (10). Similarly, an evaluation of an educational programme addressing key determinants of infant mortality in Mississippi reported significant improvements in parental knowledge, skills, and behavioural intentions post-intervention (11). An analysis of data from 1980 to 2015 in Indonesia found that maternal education programmes accounted for 15% of the decline in infant mortality, highlighting the critical role of educating mothers in improving child survival rates (12). Furthermore, breastfeeding, supported by the Baby Friendly Initiative (BFI), positively impacts multiple child health outcomes by reducing the risk of serious infections such as pneumonia and diarrhoea, which are also among the leading causes of infant mortality worldwide (14, 15).

Despite existing educational interventions and programmes, infant mortality rates remain persistently high, with significant disparities still evident, suggesting limitations in their effectiveness or reach. Additionally, cultural beliefs may hinder the impact of parenting and health education initiatives (8, 11, 16). For example, a study found that despite receiving SIDS guidance, many mothers misinterpreted or ignored it based on cultural beliefs; Pakistani-origin mothers saw it as irrelevant, while white British mothers selectively adapted it (16). Another study conducted with women in Mississippi found that participants viewed discussing topics like infant mortality as bad luck, which prevented them from applying what they had learned (i.e., health education messages), revealing a gap between knowledge and action (11). These issues underscore the need for innovative, culturally responsive interventions to bridge the gap between knowledge and action in reducing risks associated with infant mortality.

In response to this, an educational and empowerment-focused health inequality programme known as STORK was developed for vulnerable mothers, parents, families and carers whose babies are admitted to neonatal care in the West Midlands, UK (9). In this context, “vulnerable families” refers to those facing medical, social, or economic challenges, including parents of infants in neonatal care or those with limited support networks. For England, it is this cohort of hospitalised newborn babies, especially those born preterm, that are at highest risk of infant mortality (7). The STORK programme is designed to equip parents with essential knowledge and skills to reduce the risks associated with infant mortality. As described in a prior publication and on the programme's website outlining its development and early implementation, topics covered include bystander life support, choking management, recognising signs of infant illness, safe sleep practices, breastfeeding support, smoking-related risks, and access to relevant health and community services (8, 9).

The programme is typically provided during the postnatal hospital stay, either on the postnatal ward or in neonatal care settings. An initial discussion of approximately 15 minutes is held to obtain parental agreement to participate, followed by a 45–60-minute one-to-one training session delivered by a trained STORK facilitator using a digital training app for parents, family members and carers. The session is interactive and involves a combination of video demonstrations, verbal explanations, and hands-on practice (e.g., CPR and choking response techniques), allowing parents to ask questions and receive personalised guidance. While the programme is designed to be flexible and sensitive to the needs of diverse parents and families, delivery typically occurs shortly after birth, although timing may vary depending on clinical context and ward dynamics. For example, in neonatal units, parents and families may be approached while caring for a preterm infant, sometimes without prior notice. Parents are introduced to the programme by STORK facilitators and have the option to download the app to their own mobile devices for continued access. The app includes videos, guidance on emergency response, safe sleep practices, and links to relevant health services, serving as a valuable reference tool beyond the session.

In its initial roll out, the STORK programme has been widely supported by staff and parents in the region (8, 9). Further evaluation of the programme with families, carers, and facilitators is now required to provide essential insights for refining the educational programme and ultimately maximising its effectiveness in reducing risks associated with infant mortality.

Therefore, this study aims to: 1) evaluate the perceived usefulness of the STORK programme, including its accompanying digital application, for parents of preterm infants in reducing risks associated with infant mortality, to inform future implementation and refinement; and 2) explore barriers and enablers to programme delivery from the perspective of programme facilitators.

## 2 Methods

### 2.1 Study design

A descriptive qualitative study was used to explore parents' and facilitators' experiences of the STORK programme, including its perceived usefulness, acceptability, delivery in practice, and opportunities for improvement. The study is reported in accordance with the Consolidated Criteria for Reporting Qualitative Research (COREQ) guideline (Appendix 1).

### 2.2 Settings

The study was conducted at Leicester Royal Infirmary (LRI) and Leicester General Hospital (LGH), both part of the University Hospitals of Leicester (UHL) NHS Trust. UHL serves approximately 10,000 births per year in the Midlands and recorded 1,751 admissions to the Neonatal Unit (NNU) in 2024. At UHL, the STORK programme was delivered to approximately 1,053 vulnerable families in 2022 whose baby required neonatal services.

### 2.3 Participants

We used purposive sampling to identify and invite thirty parents, including mothers and fathers, who had participated in the STORK training programme. Participants included parents of infants admitted to neonatal wards at the time of recruitment/interview and parents whose infants had already been discharged. Parents were grouped according to the time since completing the STORK training: within the past four months, or more than four months and up to twelve months before the interview.

Of these, sixteen responded and participated in the study between September and December 2024, representing a response rate of 53%. Additionally, five STORK facilitators from UHL were invited via email to participate, with three responding and taking part between June and August 2024, representing a response rate of 60%. Non-response was anticipated, given the clinical and emotional demands experienced by parents of preterm infants and infants receiving neonatal care. No further recruitment was undertaken once the achieved sample was judged to provide sufficient depth and variation across both parent and facilitator perspectives, and to have generated rich data relevant to the research questions.

Participants were eligible if they were 18 years or older, able to communicate in English, and had received the STORK programme within the past four months. All participants received an information sheet and gave verbal consent before participating in the study. This consent process was approved by the UHL NHS Trust Audit and quality improvement committee as a service evaluation as the app is embedded within the Neonatal services of the Trust. Anonymity was assured by removing names and identifying details from transcripts. Participants' data were stored securely on encrypted, password-protected systems accessible only to the research team, in accordance with institutional data governance protocols. Demographic details are presented in Table 1 below. This study is reported in accordance with the COREQ (consolidated criteria for reporting qualitative research) guideline (Appendix 1).

**Table 1:** Demographics of participants.

Characteristic	Category	*n* (%)
Parents (i.e., mothers and fathers *n* = 16)		
Ethnicity	White British	9 (56%)
	Asian/Asian British	5 (31%)
	Black African	2 (13%)
Role	Mother	9 (56%)
	Father	7 (44%)
Relationship Status	Married	6 (38%)
	Cohabiting/Living with partner/Engaged	10 (62%)
Language (s) Spoken	English only	9 (56%)
	English + Other language (Gujarati, Urdu, Afrikaans)	7 (44%)
STORK Participation	Current	11 (68%)
	Completed (>4 months)	5 (31%)
**STORK Programme Facilitators (*n*** **=** **3)**		
Ethnicity	White British	2 (67%)
	Asian/Asian British	1 (33%)
Educational Level	Bachelor's degree	1 (33%)
	Master's degree	1 (33%)
Language(s) Spoken	English only	2 (67%)
	English + Gujarati	1 (33%)
Number of Sessions Conducted	30–100+	

### 2.4 Data collection

Data were collected by (OO), a social scientist with a PhD and over five years of experience conducting qualitative research, using semi-structured interviews with parents who had participated in the programme, as well as with programme facilitators. A semi-structured interview guide was designed to explore parents' experiences of the education and empowerment programme, focusing on its acceptability, perceived usefulness, barriers to engagement, and opportunities for improvement. The guide also sought to capture the perspectives of programme facilitators, including how the programme was delivered in practice and the challenges encountered during implementation. Participants were informed of these discussion areas prior to the interview. Interviews lasted 30–60 min and were conducted via telephone and Microsoft Teams for convenience, as some participants were still caring for their babies and had other childcare responsibilities. All interviews were audio-recorded with participant consent and transcribed verbatim.

### 2.5 Data analysis

We used the six-phase thematic analysis approach outlined by Braun and Clarke (2006): familiarisation with the data, generating initial codes, searching for themes, reviewing themes, defining and naming themes, and producing the report (17). Interviews were audio-recorded, transcribed verbatim, and checked for accuracy. Two researchers (OO and AS) read the transcripts several times to become familiar with the data. Coding was conducted inductively from the transcripts rather than using a predefined coding framework or topic areas from the interview guide. Initial codes were generated to capture meaningful features of the data across participants' accounts. Themes were then developed from the coded data and reviewed through iterative discussions between the researchers. Themes were refined to ensure that they represented coherent patterns of meaning across the dataset and were relevant to the study objectives. The wider research team discussed the developing analysis, and differences in interpretation were considered and resolved through discussion. Finally, the findings were written up as a coherent narrative, with each theme supported by direct participant quotations.

### 2.6 Reflexivity

Reflexivity was integrated throughout the research process to critically examine and mitigate potential biases. Prior to data collection, the researchers acknowledged assumptions that empowerment and educational programmes or interventions, could have a positive impact on parents' experiences. These perspectives and potential biases were documented in a reflexive journal and critically revisited throughout data collection and analysis to enhance transparency and reduce interpretive bias. We approached this study from a constructivist standpoint, understanding that the insights we uncovered emerged together through our conversations with each participant. Interpretations were grounded in participants' experiences. To enhance credibility, member checks, a process in which researchers share findings with participants to confirm accuracy and resonance, were conducted by another researcher (TP) to validate the emerging themes and ensure they accurately reflected participants' perspectives.

## 3 Results

### 3.1 Participant characteristics

A total of 16 parents (9 mothers and 7 fathers) participated in the study. The majority identified as White British (*n* = 9), followed by Asian/Asian British (*n* = 5) and Black African (*n* = 2). Most were in partnered relationships, including married, cohabiting, or living with a partner. Participants spoke English, with several also speaking community languages such as Gujarati, Urdu, and Afrikaans. Eleven parents were currently enrolled in the STORK programme, while five had completed it within the past four months. In addition, three STORK programme facilitators participated. They varied in ethnicity, education level, and experience, having conducted between 30 and 100 sessions. Facilitators spoke English, with one also fluent in Gujarati.

### 3.2 Overview of themes

The study findings are presented in two main sections: a) evaluating the perceived usefulness of the programme, and b) identifying barriers and facilitators to delivering the programme. Key themes generated include preferences and considerations for programme delivery, knowledge and skills development, retention of information over time, behavioural impact and changes, content of the programme, value of the app as a reference tool, hands-on training and personalized support, and language and cultural barriers in engagement. Table 2 summarises the findings and presents author-proposed recommendations for improving the programme, based on participants' accounts and the themes generated from the analysis.

**Table 2:** Key themes identified from parent and facilitator interviews exploring the STORK programme, with author proposed recommendations for programme improvement.

**Themes**	**Summary of findings**	**Recommendations**
Preferences and Considerations for Programme Delivery	Participants perceived early postpartum delivery as useful because it provided timely guidance while they were adjusting to caring for their newborn; however, they highlighted the need for flexibility, including advance notice and the option to choose a suitable session time.	Provide flexible scheduling with multiple session options. Schedule sessions soon after birth. Facilitate group sessions to promote peer support.
	Some participants also suggested that receiving the education later may improve recall, while group-based sessions were viewed as a potentially efficient and socially supportive format.	
Knowledge and Skills Development	Increased preparedness and confidence in emergency handling, safe sleep practices, and reinforced skills through hands-on practice and feedback.	Continue integrating practical, hands-on cardiopulmonary resuscitation and choking simulations. Provide immediate feedback during practice sessions.
Retention of Information Over Time	Retention was influenced by prior training experiences, session delivery methods, and fatigue levels.	Arrange periodic refresher sessions and follow-up notifications/reminders through the app. Further explore retention strategies through stakeholder/focus group discussions. Offer brief refresher videos accessible via app at any time.
Behavioural Impact and Changes	Participants reported behavioural changes, including improved infant sleep safety practices and greater awareness of risks related to smoking/vaping near infants.	Reinforce behavioural messages through app reminders. Include content explicitly addressing behavioural risks like smoking and vaping.
Content of the Programme	Participants suggested expanding content to include postpartum mental health, infant nutrition guidance, and debunking childcare myths.	Expand content to include postpartum mental health guidance and month-by-month infant nutritional guidance. Provide content addressing myths and misconceptions around childcare explicitly.
Value of the application as a reference tool	The app was valued for its clarity and as a useful reference tool, though its usage post-training varied depending on immediate needs.	Develop app functionalities like reminders, interactive scenarios, and quizzes for reinforcement. Allow interactive feedback through the app to clarify doubts and consolidate learning.

### 3.3 Perceived usefulness of the programme

#### 3.3.1 Preferences and considerations for programme delivery

One participant emphasised the need for flexibility in scheduling programme sessions, stating that having a range of available dates and times would make it easier for parents to attend, stating: “*Just having flexible options for time and date would be good. Sometimes, it's difficult to commit to a single time, especially when dealing with a newborn” (P02).* Another participant stressed the urgency of early programme intervention, noting that delaying the programme could result in missed opportunities for parents to apply the knowledge effectively: “*A couple of days after birth is ideal. If it's weeks later, anything could have happened by then, so it's good to do it early. In those early days, you're still figuring things out, and having guidance makes a difference” (P03).*

There was a noted need for better timing in programme delivery, with one participant highlighting the challenges of being approached unexpectedly for training, particularly in a neonatal setting where parents are often exhausted and preoccupied with their newborns, stating: “*I know when I had some, like, the worker come up to me, she came up to me, and she's like, ‘I'm just here to speak to you about blah, blah, blah.’ And I was like, ‘Right? I'm actually just about to feed him or just about to change him.’ My partner wasn't there, and I ended up just turning her away, saying, ‘Can you actually come back?’” (P01)*. Another participant suggested a more considerate approach, proposing that parents be given advance notice of when training will take place and the opportunity to choose a time that suits them: “*Maybe the nursery workers or the nurses on the ward could say, ‘Look, somebody's going to be coming around later today. Is there an ideal time that you and your partner feel you might be up for it?’ I think that kind of approach might work better than just somebody literally coming up to you and saying, ‘I'm here to give you this training’” (P04).*

Participants recommended shifting from one-on-one to group-based STORK sessions in hospital settings, viewing this as a more efficient, accessible, and socially supportive approach that fosters a sense of community among new parents. One participant stated: “*Maybe it would be beneficial to have sessions in the hospital where parents could attend in groups rather than one-on-one. That might save time, be useful for parents, and make it more accessible. When you're in a group, you also hear different questions and concerns, which can be really insightful*” (P05).

#### 3.3.2 Knowledge and skills development

The programme had a significant impact on participants' preparedness and confidence in handling emergency situations. One participant, reflecting specifically on the choking management training, described how their approach to such situations had changed as a result of what they had learned: “*Previously, my instinct would've been to just try and remove the object from the baby's mouth. The programme introduced a proper, step-by-step approach to handle the situation effectively” (P16).* Another participant shared that the programme increased their awareness of safe sleeping practices, particularly the importance of placing the baby at the bottom of the cot, stating: “*The recommendations have changed over the years, and now we follow the guidance on placing the baby at the bottom of the cot” (P14).*

Participants also highlighted the value of hands-on practice in reinforcing their skills. One participant noted that the STORK programme allowed them to practice CPR and choking techniques with real-time feedback, stating: “*First, we watched a video on each topic. Then, the lady explained how to give CPR correctly. My partner and I both had a go at it, and she gave us feedback on what to change and what not to change.” (P10).* Another stated: “*After the session, I know step-by-step what I should be doing in each and every step. It's critical because every second matters. I think I'm well prepared now, as compared to before the session. I was ill-prepared, and now I'm well prepared” (P09).*

#### 3.3.3 Retention of information over time

The mode of delivery was identified as a key factor influencing knowledge retention, with one participant stating that in-person sessions supported better focus and retention compared to video-based formats: “*The in-person delivery helped keep me with retaining information. If it had been a video, I don't think I would have focused as much” (P11).* Although the programme is formally designed to be delivered as a short, 15-minute face-to-face session, some participants felt that retention of information in this format was ineffective when they were physically and emotionally exhausted, with one stating: “*A 15-minute chat on the ward after three days of no sleep, no matter how good the chat was, isn't going to work.” (P13*).

Participants reported that prior experience, such as CPR training, supported their ability to recall specific aspects of the programme, with one stating: *“Yeah, I mean, I quite like the idea. I've had to rack my brain since getting the email about what I've actually retained. I remember the CPR stuff, but having prior training helps, it's not something you tend to forget” (P12).* They also emphasised that the effectiveness of the training programme should be measured not by the duration or perceived quality of the session, but by the learner's ability to recall and apply skills in real-life situations, with one stating: *“So it's not about how much time you spend. It's not even about, you know, the quality of the session itself or whether you enjoyed the session. That's not the right question. The right question is, how do you design the programme in such a way that, if I ever needed to save my baby's life, I will remember in the moment how to do it?” (P08).*

One participant noted that printed reference materials, such as handout cards or an induction pack, could serve as helpful retention aids, particularly in situations requiring immediate recall, since remembering information without prompting was challenging: *“I remember some of the cards and stuff like that. That was easy for me to recall. But without prompting, the retention is pretty small. So yeah, maybe a couple of handout cards or even an induction pack, like here are your things to do if anything goes wrong, would be quite helpful” (P11).* Another participant reflected on the challenge of ensuring long-term retention in learning experiences, emphasizing the importance of designing programmes in a way that makes knowledge stick*: “Difficult question, because to answer that question well, you have to know what you've forgotten. And if you know what you've forgotten, well, you've already forgotten it. Can I suggest something here? This might be a bit inside baseball. I'm a professional sales trainer, and one of the things that I look for when I design programmes myself is: how do we make this stick?” (P06).*

#### 3.3.4 Behavioural impact and changes

The programme encouraged behavioural changes among participants, particularly regarding smoking and vaping risks near infants, with one participant stating: “*After learning how long it takes for vapors to leave clothing, I've started changing my clothes every time I vape and waiting at least half an hour before being near the baby” (P08)*. One participant reported increased awareness of infant sleep safety, which led to proactive steps to create a safer environment: *“I'm definitely a lot more aware. Even though my baby isn't home yet, I checked the temperature in the bedroom where he'll be sleeping, set up his monitor, and checked his sleepwear to ensure everything was safe” (P06).* Another participant added: *“Now, I also check the tog ratings on my baby's bedding and sleep suits to make sure they match the room temperature” (P12).* Another reported behavioural change was the adoption of the LLF method (Look, Listen, Feel) when assessing their baby's well-being, with one participant stating: *“Now, when I see my baby still, I follow the LLF process—Look, Listen, and Feel to check if my baby is alright or not” (P13).*

#### 3.3.5 Content of the programme

While some topics were widely supported, participants expressed uncertainty about the relevance of certain content, particularly lifestyle-related discussions such as smoking cessation and general healthy living, with some questioning how well these topics fit into the programme's objectives. One stated: “*I feel like the healthy lifestyle kind of situation … it fits in a way, but it also doesn't. I feel like it's kind of like two separate parts”.* Another added: “*Yes, smoking has its contributions, but that's just a passing question. Realistically, how many parents do we know out there who say they don't smoke, or that they only smoke two cigarettes a day, when they probably don't?” (P15).* One participant stated that the programme did not highlight new risks but instead reinforced existing knowledge and practices: “*In my case, no, but, you know, just because, you know, we've got a bit of common sense around things like smoking, and, you know, we went to some antenatal classes where a lot of the same stuff was covered as well. So, in my case, it didn't highlight any new risks” (P07).*

Participants recommended expanding the programme's content to include infant nutrition, suggesting that guidance on a baby's month-by-month nutritional needs would be a valuable addition. One stated: *“Nutrition should also be included, guidance on a baby's nutritional requirements month by month would be very helpful” (P01).* Others suggested including additional information on sustaining newborn care beyond the immediate postpartum period, as well as addressing common myths about childcare, since some parents had encountered outdated or incorrect advice from informal sources. One participant stated: “*I think also there's some scope to expand the programme around the idea of, like, How do you handle those first few weeks for yourself during postpartum? It's such a blur, and you feel like you're just surviving” (P10).* Another added: “*There's stuff like—how do I put it? My wife's had a lot of bad advice, like old wives' tales … There's a lot of stuff floating around that's either useless at best or harmful at worst” (P16).*

#### 3.3.6 Value of the app as a reference tool

Participants acknowledged the app's value as a reference tool, highlighting its clarity and usefulness when needing to refresh knowledge. One participant noted: “*Yeah, it's good for reference. It's very clear and useful if we ever need it. I like that I can go back to it if I forget something” (P08).* They reported minimal use of the app after the programme, largely attributing this to not having encountered a situation that required revisiting the information, as well as the demands of caring for a newborn. One participant explained: “*Not really, as I haven't needed to. I think if something specific came up, I'd use it, but right now, everything feels manageable” (P12).* Another stated: “*I haven't had the chance to use the app because of the newborn, but I plan to revisit it when I have free time” (P11).*

They also expressed reluctance to download and use additional apps, feeling these were unnecessary since trusted information is already available online. One participant stated: “*My partner downloaded the app, but I haven't used it. I just don't want another app on my phone, quite frankly. Everything is so accessible online on the NHS website anyway, so I don't feel the need to have a specific app that does the same thing*” *(P04)*. In contrast, another participant expressed interest in revisiting the app periodically to reinforce learning and maintain preparedness: *“I would want to brush up on the whole session using the app and understand the concepts thoroughly” (P05).*

### 3.4 Barriers and facilitators to programme delivery

#### 3.4.1 Hands-on training and personalized support

The programme placed a strong emphasis on hands-on training to ensure families, parents and carers felt confident handling emergency situations, with one facilitator noting that, based on her interactions with most parents, they found the life support and choking prevention training particularly beneficial: “*Choking and breathing emergencies are a parent's worst nightmare. The training gives them the confidence to act in those critical moments*” *(F1).* Training dolls were used with parents to reinforce learning and provide realistic practice, while facilitators emphasised the importance of expanding hands-on training. One facilitator shared: “*We also used dolls for hands-on practice, making the training even more impactful*” *(F2).* Another added: *“I plan to continue refining my hands-on training skills and gaining more experience to improve my ability to engage and educate parents” (F3).*

Facilitators used an individualized approach to deliver the programme, ensuring parents understood the content by guiding them through the app, answering questions, and providing refresher resources. One facilitator explained: “*I introduce myself, explain the programme, and often wait for partners or family members to join. I go through the app with them, explain topics, answer questions, and ensure they have access to a refresher afterward*” *(F1).*

#### 3.4.2 Language and cultural barriers in engagement

A key challenge in delivering the programme was overcoming language barriers. While Language Line was initially used, it was time-consuming, so multilingual facilitators and neonatal nurses helped with translation. One facilitator explained: “*Initially, there were language barriers. We had to use Language Line for interpretation, which was time-consuming. Later, we employed facilitators who spoke different languages, and neonatal nurses also assisted” (F3).* Cultural differences also shaped how some parents engaged with the programme, particularly in relation to infant sleep safety. Facilitators described needing to explain UK safe sleep guidance clearly and respectfully where parents were familiar with sleep practices that differed from this guidance. Another facilitator shared*: “Cultural differences were another challenge, as many parents followed different sleeping practices, and we had to explain UK guidelines clearly and respectfully” (F2).*

#### 3.4.3 Resource constraints impacting training delivery

Another significant challenge was the shortage of facilitators, which limited the programme's ability to provide one-on-one training to every parent. As a result, group sessions were suggested as a viable way to increase reach and accessibility. A facilitator emphasised this concern: “*More facilitators are needed. Every parent should have access to this training. Group sessions might also help reach more parents” (F3).* Additionally, interpreter shortages and the absence of pre-translated materials posed further barriers for non-English-speaking parents. One facilitator noted: “*Yes, mainly interpreter shortages and the absence of pre-translated materials. Google Translate was sometimes inaccurate” (F1)*.

## 4 Discussion

Our study aimed to evaluate the perceived usefulness of the STORK programme among parents, families and carers of preterm infants, and to explore perceived barriers and facilitators to its delivery from the perspective of programme facilitators, with findings from both groups informing future improvement, implementation and delivery of the programme. Participants preferred hands-on, flexible, and timely delivery, with some suggesting that receiving the education later could increase recall, and group sessions were also suggested for efficiency and peer support. While some lifestyle topics (e.g., smoking cessation) were questioned, parents valued the practical content and requested additional information, such as infant nutrition and myth-busting, to be embedded within the programme. STORK facilitators highlighted barriers to programme delivery, including challenges in engagement due to cultural and language differences, as well as a shortage of facilitators to provide one-on-one training to parents, families, and carers. They suggested increasing the number of facilitators and incorporating group-based models to improve programme delivery.

Participants reported increased confidence in handling emergency situations after gaining knowledge and skills from the training programme. This aligns with similar findings from other infant safety training initiatives. In a survey of 375 parents similarly showed that those with prior infant basic life support (IBLS) training scored higher on knowledge tests (median score 8/9 vs. 6/9 for untrained parents) and were far more likely to achieve a passing knowledge score (18). In another study, parents in a U.S. neonatal intensive care unit (NICU) who used a self-instruction CPR kit reported a significant increase in their confidence performing infant CPR over 12 months (from a baseline score of 2.8–3.5 out of 5) and felt better prepared to respond in an emergency after training and practice sessions (19). These studies reinforce that formal training can improve either parental knowledge or confidence in responding to infant emergencies.

A critical question for any training programme is whether participants remember and apply these life-saving details when needed, even months later. Studies assessing long-term retention in similar programmes reveal mixed findings and highlight the need for refreshers (14, 20). For example, a study measured parents' CPR performance six months after initial training: alarmingly, only about one-third (33.7%) of parents could still perform infant CPR correctly at the 6-month mark, indicating substantial skills decay over time (21). In another study, a safe-sleep education intervention found that parents retained key knowledge, such as the protective effect of pacifier use against SIDS, with significantly improved understanding persisting 10 weeks after training, indicating effective short-term knowledge retention (20). Parents recommended monthly refresher sessions to enhance the retention of life-saving knowledge and skills, acknowledging that confidence to act may decline over time without regular practice (18, 22). Our findings echoed these concerns. Participants felt that in-person sessions supported focus and memory better than video formats, and some found printed materials helpful for recall. However, fatigue and the hospital environment were seen as barriers to retention. To improve this, participants suggested follow-up resources such as handout cards or induction packs and emphasised the need for program design that supports recall in high-stress situations.

Our study, along with others, documented self-reported behavioural changes among participants in the STORK programme and other similar educational initiatives. A study reported safer sleep practices among parents who received safe sleep education, with follow-up showing an increased likelihood of placing infants in a supine position, using a pacifier at bedtime, and avoiding loose bedding (20). Previous research suggests that community-based interventions combining safe sleep education with the provision of appropriate material resources, such as bassinets or portable play yards, can support adherence to recommended infant safe sleep practices (23). Additionally, incorporating CPR practice into routine pediatric visits has been proposed to enhance parental readiness (16). Sustaining these behaviours can be challenging. One study found that parents who initially follow safe sleep recommendations often revert to less safe practices, such as bedsharing due to fatigue, when their infant wakes at night, highlighting the need for practical strategies to support long-term behavior change (24). Given that bedsharing is a common coping strategy used by parents to balance their own needs with those of their infants, some approaches advocate offering harm-reduction guidance, such as how to minimise risks when bedsharing does occur, rather than promoting strict avoidance. This aligns more closely with UK policy, which differs from abstinence-focused US guidelines, and may be more effective in real-world settings. Additionally, combining education with tangible resources or policy efforts drives greater behaviour change. For example, a child passenger safety programme found that, while education alone had some impact, providing a free car seat and installation training significantly increased usage three months postpartum, thereby improving driving safety behaviours (25).

Participants suggested expanding the programme to include infant nutrition guidance, strategies for managing the postpartum period, and debunking common childcare myths.​ A similar study exploring parents' perceptions of health promotion found that while parents recognize the importance of healthy lifestyle discussions, they often question their relevance within parenting programmes, suggesting a need for more tailored content (26). Research into family-based lifestyle interventions highlighted that participants value practical strategies and interpersonal support over general lifestyle advice, emphasizing the importance of interactive and engaging content (27). Addressing the postpartum period and debunking childcare myths is crucial, as research shows parents seek early parenting support and accurate information to counter misinformation (27). Aligning programme content with participants' perceived needs, such as focusing on practical infant care and nutrition while addressing lifestyle factors in a contextually relevant manner, may enhance engagement with educational programmes.

Our study findings, along with those of others, have shown that mobile apps have emerged as a complementary strategy for parental education, though evidence of their impact is still developing ​ (28–30). For example, a study reported that a mobile app supported remote monitoring and counselling for high-risk pregnancies, maintained care with high patient compliance, and did not increase adverse outcomes ​ (28). Likewise, smartphone messaging services (e.g., Text4Baby in the US) have been used to deliver tips on safe sleep, breastfeeding, and recognizing warning signs, demonstrating improvements in parental knowledge and engagement (29). Thus, while technology can broaden reach and tailor messages, it is generally seen as an adjunct to, not a replacement for, traditional education and support (30). The consensus is that multi-modal approaches (combining online education, print materials, and person-to-person counselling) work best to reinforce critical public health messages (31). To further enhance engagement following the delivery of the STORK programme, future iterations could include personalised push notifications aligned with infant developmental stages, interactive tracking features (e.g., breastfeeding or vaccination logs), and peer support forums to foster ongoing connection and support. These enhancements could help sustain parental engagement beyond the structured sessions, offering timely and personalised support as caregiving needs evolve.

## 5 Implication for future research

Future research should explore how to optimise the delivery and scalability of STORK as a public health educational programme within a diverse healthcare setting. This includes investigating the effectiveness of different delivery formats, such as group-based sessions, digital platforms, or hybrid models, in enhancing accessibility and engagement. Research should also consider the role of cultural and linguistic adaptation in improving inclusivity and comprehension across varied populations. Additionally, there is value in examining strategies that promote long-term knowledge retention and behaviour change, particularly in high-stress, real-life scenarios. Understanding how digital tools can be better integrated into routine care to support learning and recall remains an important area for exploration, alongside evaluating the feasibility and cost-effectiveness of broader programme implementation across maternity and neonatal care services.

## 6 Study strengths and limitations

The inclusion of participants with diverse ethnic and linguistic backgrounds enhanced the study's relevance to multicultural healthcare settings. Conducting the research across two major hospitals serving high-risk populations also supports transferability. However, limitations include a relatively small sample size, limited to English-speaking participants, which may exclude perspectives from non-English speakers. Additionally, potential selection bias may have occurred, as those more engaged with the programme may have been more likely to participate in the study. The study also did not capture the age of babies at the time of interview, meaning it was not possible to determine whether participants were interviewed during the period when purple crying or ICON messages may have been especially relevant. This may have influenced participants perceived need to revisit or use the programme content after completion.

## 7 Conclusion

The evaluation of the STORK programme suggests that it is a well-accepted and practical parent education programme. Parents perceived the programme to improve their confidence and preparedness for infant care, particularly in responding to emergencies through CPR and choking training. They also reported increased awareness and self-reported behaviour changes relating to safe sleep and smoke exposure. While this study did not assess mortality outcomes directly, the findings suggest that brief, appropriately timed education may support parents in recognising and addressing modifiable risks. Given its acceptability and perceived benefits, STORK may offer value relative to the time and resources required for delivery; however, this has yet to be formally determined, as a detailed cost analysis and health economic assessment were not conducted. The need for greater cultural tailoring was also identified as a key consideration for future implementation and scale-up, to ensure the programme is accessible, relevant and effective for diverse populations.

## Data Availability

The raw data supporting the conclusion of this article will only be provided upon reasonable request.

## Appendix 1. COREQ Checklist.

**Table:**

No. Item	Guide questions/description	Reported on Page #
Domain 1: Research team and reﬂexivity		
*Personal Characteristics*		
1. Inter viewer/facilitator	Which author/s conducted the interview or focus group?	Page 5
2. Credentials	What were the researcher's credentials? E.g. PhD, MD	Page 5
3. Occupation	What was their occupation at the time of the study?	Page 5
4. Gender	Was the researcher male or female?	Page 5
5. Experience and training	What experience or training did the researcher have?	Page 5
*Relationship with participants*		
6. Relationship established	Was a relationship established prior to study commencement?	Nil
7. Participant knowledge of the interviewer	What did the participants know about the researcher? e.g., personal goals, reasons for doing the research	Page 5
8. Interviewer characteristics	What characteristics were reported about the inter viewer/facilitator? e.g., Bias, assumptions, reasons and interests in the research topic	Page 7
Domain 2: study design		
Theoretical framework		
9. Methodological orientation and Theory	What methodological orientation was stated to underpin the study? e.g., grounded theory, discourse analysis, ethnography, phenomenology, content analysis	Page 7
Participant selection		
10. Sampling	How were participants selected? e.g., purposive, convenience, consecutive, snowball	Page 5
11. Method of approach	How were participants approached? e.g., face-to-face, telephone, mail, email	Page 5
12. Sample size	How many participants were in the study?	Page 5
13. Non-participation	How many people refused to participate or dropped out? Reasons?	Page 5
Setting		
14. Setting of data collection	Where was the data collected? e.g., home, clinic, workplace	Page 7
15. Presence of non-participants	Was anyone else present besides the participants and researchers?	No
16. Description of sample	What are the important characteristics of the sample? e.g., demographic data, date	Page 6
Data collection		
17. Interview guide	Were questions, prompts, guides provided by the authors? Was it pilot tested?	Page 6
18. Repeat interviews	Were repeat inter views carried out? If yes, how many?	No
19. Audio/visual recording	Did the research use audio or visual recording to collect the data?	Page 7
20. Field notes	Were ﬁeld notes made during and/or after the interview or focus group?	No
21. Duration	What was the duration of the interviews or focus group?	Page 7
22. Data saturation	Was data saturation discussed?	No
23. Transcripts returned	Were transcripts returned to participants for comment and/or correction?	No
Domain 3: analysis and ﬁndings		
Data analysis		
24. Number of data coders	How many data coders coded the data?	Page 7
25. Description of the coding tree	Did authors provide a description of the coding tree?	Page 7
26. Derivation of themes	Were themes identiﬁed in advance or derived from the data?	Page 7
27. Software	What software, if applicable, was used to manage the data?	No
28. Participant checking	Did participants provide feedback on the ﬁndings?	Page 7
Reporting		
29. Quotations presented	Were participant quotations presented to illustrate the themes/ﬁndings? Was each quotation identiﬁed? e.g., participant number	Page 8 to 14
30. Data and ﬁndings consistent	Was there consistency between the data presented and the ﬁndings?	Page 8 to 14
31. Clarity of major themes	Were major themes clearly presented in the ﬁndings?	page 8 to 14
32. Clarity of minor themes	Is there a description of diverse cases or discussion of minor themes?	No
