# Correction: Evaluating a neonatal public health education and empowerment programme to reduce infant mortality risks: insights from parents and facilitators in the midlands, UK

**DOI:** 10.3389/fgwh.2026.1947483

**Published:** 2026-08-17

**Authors:** Olufisayo Olakotan, Samuel Oluwatobi Atiku, Gregory Maniatopoulos, Nicola O’Brien, Thillagavathie Pillay

**Affiliations:** 1 Department of Neonatology, Women and Children’s Directorate, University Hospitals Leicester NHS Trust, Leicester, United Kingdom; 2 School of Digital, Technology, Innovation and Business, University of Staffordshire, Stafford, United Kingdom; 3 Research Data and Systems Manager, Aston University, Birmingham, United Kingdom; 4 School of Business, University of Leicester, Leicester, United Kingdom; 5 School of Psychology, Northumbria University, Newcastle upon Tyne, United Kingdom; 6 Faculty of Science and Engineering, University of Wolverhampton, Wolverhampton, United Kingdom; 7 College of Health Sciences, University of Leicester, Leicester, United Kingdom

**Keywords:** health inequalities, infant mortality, parent education, parental empowerment, preterm infants, STORK programme

There was an error in the description of the delivery of the STORK programme in the **Introduction** section. The published text incorrectly described the programme as a single short, approximately 15-minute, face-to-face session.

The original text read:

*“The programme is typically delivered during the postnatal hospital stay, either on the postnatal ward or in neonatal care settings, through a single short (approximately 15-min) face-to-face session led by trained STORK facilitators using a digital training app, delivered one-on-one with parents, family members and carers.”*

A correction has been made to the section **Introduction**, paragraph 5:

*“The programme is typically provided during the postnatal hospital stay, either on the postnatal ward or in neonatal care settings. An initial discussion of approximately 15 min is held to obtain parental agreement to participate, followed by a 45–60-minute one-to-one training session delivered by a trained STORK facilitator using a digital training app for parents, family members and carers.”*

This correction does not change the scientific conclusions of the article in any way.

The original version of this article has been updated.

